# The association between material-psychological-behavioral framework of financial hardship and markers of inflammation: a cross-sectional study of the Midlife in the United States (MIDUS) Refresher cohort

**DOI:** 10.1186/s12889-023-16745-x

**Published:** 2023-09-21

**Authors:** Agus Surachman, Reginald Tucker-Seeley, David M. Almeida

**Affiliations:** 1 Department of Epidemiology and Biostatistics, Dornsife School of Public Health, Drexel University, Philadelphia, 19104 USA; 2 College of Nursing and Health Professions, Drexel University, Philadelphia, 19104 USA; 3 ZERO – The End of Prostate Cancer, Alexandria, VA 22314 USA; 4 Department of Human Development and Family Studies, The Pennsylvania State University, University Park, PA 16802 USA; 5 Center for Healthy Aging, The Pennsylvania State University, University Park, PA 16802 USA

**Keywords:** C-reactive protein, Fibrinogen, Financial hardship, Household economic well-being, Inflammation, Interleukin-6, Material–psychological–behavioral domain

## Abstract

**Background:**

Measures of financial hardship have been suggested to supplement traditional indicators of socioeconomic status (SES) to elucidate household economic well-being. This study formally tested the construct validity of financial hardship and examined its association with markers of inflammation.

**Methods:**

This study utilized data from the Midlife Development in the United States Refresher Study (MIDUS-R; Age = 23-76, 53.7% female, 71% white). Participants were divided into exploratory factor analysis (EFA; completed SAQs only; *N* = 2,243) and confirmatory factor analysis sample (CFA; completed SAQs and biomarker assessment; *N* = 863). Analysis was divided into three steps. First, exploratory factor analysis (EFA) is used to examine if the three-domain factor (material, psychological, and behavioral) is the best fitting model for financial hardship measures. Second, we conducted CFA to test the hypothesized three-factor measurement model of financial hardship. Third, we tested the association between domains and the general latent factor of financial hardship and inflammation (interleukin 6/IL6, c-reactive protein/CRP, and fibrinogen).

**Results:**

Results from EFA supported the three-domain model of financial hardship. The hypothesized three-domain measurement model fits well in a different sample within MIDUS-R. In the models adjusted for age and sex, higher material hardship was associated with elevated IL6, CRP, and fibrinogen, while higher behavioral hardship was associated with higher CRP. The association between the material domain and IL6 remained significant after adding body mass index, education, and race as additional covariates. The second-order financial hardship measurement model was associated with IL6, CRP, and fibrinogen, adjusted for age, sex, BMI, education, and race.

**Conclusion:**

Explicating the socioeconomic environment to include indicators of financial hardship can help researchers better understand the pathway between SES and the inflammation process, which may help elucidate pathways between SES and age-related chronic diseases associated with inflammation.

**Supplementary Information:**

The online version contains supplementary material available at 10.1186/s12889-023-16745-x.

## Background

Research has consistently shown that adverse socioeconomic circumstances (e.g., low educational attainment) negatively affect health and well-being [1]. One line of this research explores mechanisms through which these adverse social circumstances “get under the skin” using measures of inflammatory processes [2, 3]. This research has shown that lower socioeconomic status is associated with elevated inflammatory markers such as C-reactive protein (CRP) [4–8], interleukin-6 (IL6) [5, 7, 8], and fibrinogen [6, 9, 10]. Inflammation is a complex biological response of the immune system [11], and it is the dysfunction of this complex system that can lead to chronic inflammation [12]. The role of chronic inflammation in aging and age-related diseases has been explored in several large epidemiologic studies [13]. In particular, these studies have shown that chronic inflammation is associated with cardiovascular disease [14], cancer [15], diabetes [16, 17], and Alzheimer’s disease [18].

Socioeconomic status is often conceptualized in the research literature as a multidimensional construct [19], usually operationalized using one (or two) traditional measures (e.g., income or educational attainment) [20]. Determining the influence of socioeconomic circumstances not captured by these traditional indicators of individual socioeconomic status on health may help further explain the pathways leading to racial/ethnic and socioeconomic health disparities [21]. Calls have been made in the social determinants of health literature suggesting a need for using measures of socioeconomic circumstances that go beyond traditional measures to better capture how SES is lived [22]. Measures of financial hardship have been proffered as useful indicators for household economic well-being [23]. Yet, there is no agreed-upon definition or measure of financial hardship across research fields. However, cancer researchers recently developed a three-domain conceptual model to help sort the many terms used to capture the financial hardship experience in the context of cancer survivorship [24–26]. The three-domain conceptual model is characterized by: 1) a *material domain* that captures the lack of financial resources, 2) a *psychological domain* that captures how people feel about their lack of financial resources, and 3) a *behavioral domain* that captures how people manage (or adjust) their financial resources or the purposeful efforts that people use to economize to meet their financial obligations. Sorting the many terms used to describe the financial hardship experience into the *material, psychological*, and *behavioral* domains is informed by the materialist, psychosocial, and behavioral explanations for health inequities [27]. Research has yet to explore the validity of this construct and whether this approach to financial hardship can be useful to elucidate socioeconomic health disparities above and beyond the traditional measures of SES.

While neither the causal pathway from financial hardship to health outcomes nor how financial hardship contributes to health disparities have been fully explicated, the influence of the material, psychological, and behavioral aspects of socioeconomic circumstances on health have been described using cognitive load and stress-related theories and theories of health disparities. Specifically, cognitive load theory suggests that poor cognitive performance can result from the attentional capture that material hardship causes; that is, the material financial hardship experience consumes cognitive resources, thus limiting information processing capacity [28]. The conservation of resources stress theory suggests that the depletion of financial resources or even the threat of the depletion of resources where individuals may struggle to meet their financial obligations can cause financial stress, which describes the psychological domain of financial hardship [29, 30]. Material, psychological, and behavioral pathways have been used to explain socioeconomic disparities in health [31, 32], suggesting that material factors shape the psychological resources and health behaviors of individuals [33]. Specifically, materialist explanations of health disparities have shown that differences in basic needs such as food, housing, and access to services and amenities contribute to disparities in health [27].

A few studies have explored the association between financial hardship and physiologic outcomes [34, 35]. However, to our knowledge, no study has explored the material, psychological, and behavioral aspects of financial hardship on physiologic responses (e.g., markers of inflammation). To address these gaps in the research literature, the goals for the current study were twofold. First, we investigated the factor structure and validity of the items related to financial hardship and examined if they fit the material-psychological-behavioral framework of financial hardship. Second, we investigated the association between the material-psychological-behavioral framework of financial hardship and multiple indicators of inflammation among US adults.

## Methods

### Data and participants

We used data from the Midlife Development in the United States (MIDUS), a national study of health and well-being (http://midus.wisc.edu). The MIDUS study was started in 1995 (MIDUS 1), followed by the longitudinal follow-up (MIDUS 2) in 2004. Descriptions of the MIDUS 1 and 2 studies are provided elsewhere [36, 37]. In 2011, a new national probability sample was recruited to participate in the MIDUS Refresher study. The MIDUS Refresher study aimed to refresh and expand the overall MIDUS study by recruiting a new sample comparable to the participants in MIDUS 1 in terms of demographic characteristics. In addition, the MIDUS Refresher was intended to examine the impact of the Great Recession in the late 2000s on health and well-being [38, 39].

The study protocol in the MIDUS Refresher was the same as in the previous waves of MIDUS, in which participants were recruited through random dial digits and completed a 30-min phone interview (*n* = 3,577, response rate 59%). Most MIDUS Refresher participants who completed the phone interview (73%) also completed self-administered questionnaires (SAQs). To increase the racial diversity of participants in the MIDUS Refresher, a supplemental sample comprising most Black participants was recruited from Milwaukee County, WI. The Milwaukee supplemental sample included 508 (response rate = 47.7%) participants who completed in-person interviews; 59% of the participants also completed SAQs. Most of the questions regarding financial hardship were part of the SAQs. In addition, only participants who completed SAQs were eligible to complete the biomarker data collection in the MIDUS Refresher. Thus, only participants who completed SAQs were included in the analytic sample (*N* = 3,106). Participants who completed SAQs but did not complete the biomarker data collection were included in the exploratory factor analysis or EFA (*N* = 2,243). The remaining participants who completed SAQs (*N* = 863) completed the biomarker study and were included in the confirmatory factor analysis (CFA). The EFA and CFA samples were similar in sociodemographic characteristics, including age, sex, and marital status (Table 1). Relative to the EFA sample, the CFA sample included fewer Black adults and more participants with bachelor’s degrees or higher (Table 1).

**Table 1:** Descriptive statistics for participants’ sociodemographic characteristics, financial hardship, and biological markers

**Sociodemographic**	%, *M*, *SD*		|*t*| or |χ|
	EFA Sample (*N* = 2,243)	CFA Sample (*N* = 863)	
Age (*M*, *SD*; range)	50.62 (14.51)	50.84 (13.41)	0.41
Female (%)	54.3	52.1	1.17
Race			
White (%)	71.1	70.6	8.42 ^*^
Black (%)	21.2	18.8	
Others (%)	7.6	10.6	
Non-Hispanic	96.2	96.1	0.04
Married (%)	57.7	58.5	0.16
Education			
HS/GED or lower	29.9	17.3	61.7 ^***^
Some college	30.8	30.5	
Bachelor’s degree or higher (%)	39.3	52.2	
**Financial Hardship**			
**Material Domain**			
Income to poverty line ratio			
< 300% (%)	44.3	35.4	21.37 ^***^
≥ 300%- < 600% (%)	30.2	32.7	
≥ 600% (%)	25.6	31.9	
No health insurance coverage (%)	11.8	9.2	4.22 ^*^
Received public/government assistance last calendar year (%)	32.1	29.6	1.79
**Psychosocial Domain**			
Perceived current financial situation (*M*, *SD*)	5.81 (2.42)	5.95 (2.46)	1.41
Perceived financial control (*M*, *SD*)	6.20 (2.70)	6.34 (2.70)	1.32
Perceived Availability of money to meet needs			
More money than need (%)	32.7	29.9	3.92
Enough money (%)	50.8	50.1	
Not enough money (%)	16.4	20.0	
Perceived difficulty paying monthly bills			
Not at all difficult (%)	11.0	10.6	3.92
Not very difficult (%)	31.6	29.3	
Somewhat difficult (%)	30.4	29.7	
Very difficult (%)	27.0	30.4	
**Behavioral Domain**			
Missed a credit card payment (%)	15.4	16.9	1.07
Missed other debt payment (%)	14.5	14.7	0.02
Sold possessions to make ends meet (%)	23.0	22.8	0.02
Cut back on spending (%)	74.6	73.9	0.16
**Physiological Markers**			
**Inflammation Markers**			
*ln* Interleukin 6 (IL6; pg/mL; *M*, *SD*)	—	0.72 (0.80)	—
*ln* C-Reactive Protein (CRP; ug/mL; *M*, *SD*)	—	0.50 (1.28)	—
Fibrinogen (g/L; *M*, *SD*)	—	3.43 (0.74)	—
**Health-related Covariates**			
BMI (Kg/m^2^; *M*, *SD*)	—	30.59 (9.54)	—
Number of chronic conditions	—	4.29 (3.40)	—
Using prescription drugs (%)	—	70.7	—

*M *Mean, *SD *Standard deviation, *ln* natural log, *|t| or |χ|* t-statistic or chi-squared value from comparing EFA and CFA samples

* = *p* < .05, *** = *p* < .001

### Analytic sample

#### ***EFA sample (N*** = ***2,243)***

The EFA sample was 54.3% female, with a mean age of 50.62 (*SD* = 14.51, range = 23–75). Most self-identified as white (71.1%) and non-Hispanic (96.2%). Almost one-third of the EFA sample (29.9%) completed HS/GED or less, and more than half (57.6%) are currently married.

#### ***CFA sample (N*** = ***863)***

 The CFA sample included participants who completed both SAQs and the biomarker assessment in the MIDUS Refresher study. The biomarker assessment was conducted from 2013 to 2016. Participants completed the biomarker assessment six months to 2 years after completing the baseline survey, with a follow-up time median of about one year. Participants were invited to stay overnight at one of the three regional clinical research units (CRUs) on the East, Midwest, and West Coasts. The CRU selection for each participant was based on the one that imposed the least travel burden. Participants completed comprehensive biological and health assessments during the stay, including blood and urine sample collections. The CFA sample was 52.1% female, with a mean age of 50.84 (*SD* = 13.41, range = 25–76). More than half (58.5%) are married, and almost one-fifth completed HS/GED or less. The EFA sample was predominantly white (70.6%) and non-Hispanic (96.1%).

## Measures

### Financial hardship

Data regarding financial hardship were collected during the baseline survey (2011–2014). Financial hardship was assessed using 11 indicators in the baseline survey. The authors sorted these items into the material, psychological, and behavioral domains previously described in the cancer prevention and survivorship research [24].

#### Material domain

*The material* domain includes indicators related to the lack of financial resources [24]. Three indicators were used as the measures of the material domain of financial hardship: 1) *income to poverty line ratio*, adjusted for the total household size (3 =  < 300%, 2 =  ≥ 300% but less than 600%, 1 =  ≥ 600%), 2) *health insurance coverage* (no = 1 or yes = 0), and 3) *public/government financial assistance*, based on whether the household received public or government assistance in the last calendar year (yes = 1, or no = 0), such as supplemental security income, social security disability insurance, food stamps, Temporary Assistance for Needy Family (TANF), and unemployment benefits.

#### Psychological domain

As part of the psychological domain, we included two indicators related to perceived financial satisfaction and two measures related to perceived financial stress or worry. Measures of perceived financial satisfaction include *current financial situation* and *financial control*. Participants reported their current financial situations on a 0 (“the worst possible financial situation”) to a 10 (“the best possible financial situation”) scale. To operationalize financial control, we used the item that asked participants: “How would you rate the amount of control you have over your financial situation these days?” on a 0 (“no control at all”) to 10 (“very much control”) scale. The original scores for the current financial situation and financial control were reverse-coded. Thus, higher scores represent higher financial hardship. Measures of perceived financial stress or worry include: 1) *perceived availability of money to meet needs* and 2) *perceived difficulty paying bills*. To operationalize the availability of money to meet needs, we used the item that asked participants: “In general, would you say you (and your family living with you) have more money than you need, just enough for your needs, or not enough to meet your needs?” on a 1–3 scale (3 = not enough money, 1 = just enough money, 1 = more money than you need). To operationalize the difficulty level of paying bills, we used the item that asked participants to rate their difficulty level in paying monthly bills on a 1–4 scale (4 = very difficult, 3 = somewhat difficult, 2 = not very difficult, 1 = not at all difficult).

#### Behavioral domain

Measures for the behavioral domain were taken from a scale used in the National Survey of Unemployed Adults conducted by the Heidrich Center for Workforce Development that included job-, home-, and financial-related hardships [40]. For this analysis, we included four items related to behavioral actions associated with dealing with financial hardship [39]. Participants responded to whether they ever experienced the following (1 = Yes, 0 = No): 1) missed a credit card payment, 2) missed other debt payment (e.g., car or student loans), 3) sold possessions to make ends meet, and 4) cut back on spending.

### Inflammation markers

We included three inflammation markers in this analysis, interleukin 6 (IL6), c-reactive protein (CRP), and fibrinogen. Inflammation markers were assayed from fasting blood samples collected before breakfast on the second day of biomarker assessment. Blood samples were collected using standardized procedures Field [39] to ensure consistency. IL6 was measured using the Quantikine® High-sensitivity ELISA kit #HS600B (R & D Systems, Minneapolis, MN). The assay range was 0.156–10 pg/mL, intra-assay CV was 3.73%, and inter-assay CV was 15.66%. CRP was measured using a particle-enhanced immunonephelometric assay (BNII nephelometer, Dade Behring Inc., Deerfield, IL). The assay range was 0.014–216 ug/mL, intra-assay coefficients of variability (CVs) ranged from 2.2 to 4.1%, and inter-assay CVs ranged from 4.72 to 5.16%. Finally, fibrinogen antigen was measured using the BNII nephelometer (N Antiserum to Human Fibrinogen; Siemens, Malvern, PA). The assay range was 2.8–4560 mg/dL, intra-assay CV was 2.7%, and inter-assay CV was 4.13–6.64%. IL-6 was assayed in the MIDUS Biocore Laboratory at the University of Wisconsin, Madison, WI. CRP and Fibrinogen were assayed at the Laboratory for Clinical Biochemistry Research at the University of Vermont, Burlington, VT.

### Covariates

Sociodemographic, SES and health-related factors were included as covariates, including age (years), sex (0 = female, 1 = male), body mass index (BMI; kg/m^2^), education (0 = no bachelor’s degree, 1 = bachelor’s degree or higher), and race/ethnicity (0 = racial/ethnic minority, 1 = non-Hispanic white).

### Analysis

Analysis for this study was conducted using a structural equation modeling (SEM) framework and divided into three steps. Pre-analysis steps for SEM were performed, including extensive data assessment, cleaning, and missing data analysis [38, 41]. Data were inspected for the potential univariate (through standardized scores, |z|≥ 3.30) and multivariate (i.e., Mahalanobis Distance *p* < 0.001 and Studentized Deleted Residual greater than ± 4.00) outliers. Although few univariate outliers were identified, we retained them as they were minimally severe (less than four standard deviations away from the mean) [42]. Furthermore, no multivariate outliers were found. Due to normality concerns, natural log-transformed (*ln*) data for CRP and IL6 were used for analysis. The assessment of model fit and accuracy in all steps of the analysis was based on multiple criteria, including [43, 44]: (a) various fit indices to evaluate overall goodness of fit, (b) examining whether there were concentrated areas of strain in the solution, and (c) size of the estimates, statistical significance, and the interpretability of the model’s parameter estimates. Given the mixture of continuous, categorical, and binary financial hardship items, we utilized the weighted least squares with mean and variance adjustment (WLSMV) estimation method. We reported the standardized estimates with a 95% confidence interval (*CI*). Most of the analysis was conducted in Mplus 8.8 [45].

### Step 1: Exploratory Factor Analysis (EFA)

We conducted exploratory factor analysis (EFA) involving the EFA sample to test whether each item of financial hardship mapped into the corresponding hypothesized domains (material, psychological, and behavioral). Domains of financial hardship were hypothesized to be correlated with each other. Thus, we utilized an oblique rotation method. Given the strong theoretical foundation of the three-factor solution for financial hardship, we used CF- FACPARSIM rotation to minimize factor complexity by spreading variances evenly across all rotated factors [46]. Only current financial status and control over financial status were treated as continuous variables, while the rest were treated as categorical variables (using the CATEGORICAL command in Mplus). We tested one-, two-, three-, and four-factor models in the analysis based on the polychoric correlation matrix with the WLSMV estimation method. We hypothesized that the three-factor model would show a better fit relative to the other solutions. We also examined additional factor retention criteria to evaluate the number of factors, including parallel analysis based on the 95th percentile of random eigenvalues, the Empirical Kaiser criterion, the Hull method, and comparison data. We hypothesized that in the three-factor model, financial hardship questions from the same domain would show higher loadings on the same factor. We utilized the factor loading cutoff of |0.4| to consider whether the question was meaningfully associated with the corresponding factor [47].

### Step 2: Confirmatory Factor Analysis (CFA)

Confirmatory factor analysis (CFA) was conducted to test the hypothesized three-factor model of financial hardship in a different sample from the MIDUS Refresher study (i.e., the CFA model). We compared the three-factor to the one-factor measurement model. Since WLSMV was used, the chi-square difference test between the one-factor vs. three-factor measurement model was conducted using the DIFFTEST command in Mplus based on the formula developed by Satorra and Bentler [48].

### Step 3: testing the association between financial hardship and inflammation

The third step of the analysis was to examine if the three hypothesized latent factors of financial hardship were associated with IL6 and CRP among adults in the CFA sample. Prediction of IL6 and CRP by financial hardship was conducted in separate models. The marker of inflammation was also regressed on covariates to account for the influence of key sociodemographic and health-related factors (MODEL 1 = adjusted for age and sex; MODEL 2 = adjusted for age, sex, and BMI; MODEL 3 = adjusted for age, sex, BMI, education, and race). Finally, we tested the association between the second-order measurement model of financial hardship (with three latent domains in the first order) and all the inflammatory biomarkers, controlling for the covariates. The model fit of the second-order measurement model would be identical to the model fit of the hypothesized CFA model because we have three factors in the first order (i.e., the higher-order factor identification was *just identified*).

## Results

### Descriptive statistics

Descriptive statistics for the financial hardship variables and the inflammation markers are presented in Table 1. Almost all indicators of financial hardship were similar between EFA and CFA samples, except for income to poverty line ratio (more participants within the < 300% category in the EFA sample) and health insurance coverage (more participants with no coverage in the EFA sample), both are part of the material domain of financial hardship. Information regarding inflammation and health-related covariates is only available from the CFA sample (i.e., MIDUS Refresher Biomarker study participants). Bivariate polychoric correlation among the financial hardship measures (for the EFA sample) and financial hardship measures and inflammation markers (for the CFA sample) are presented in Supplementary Material 1.

### Exploratory Factor Analysis (EFA)

We hypothesized that measures of financial hardship would follow the three-domain factor solution, namely material, psychological, and behavioral. The analysis included a sufficient sample size (*N* = 2,243) and participant-to-item ratio (172.5 to 1) for stable and replicable factor solutions. Our data showed a meritorious Kaiser–Meyer–Olkin (KMO) value of 0.89. Bartlett’s test of sphericity was significant (χ^2^ [55] = 13,215.03, *p* < 0.001), indicating that the data are suitable for factor analysis. Results from EFA suggested that the 3-factor model was the best-fitting solution (χ^2^ = 125.88, *df* = 25, *p* < 0.001; *RMSEA* = 0.04; *CFI* = 0.99; *TLI* = 0.98; *SRMR* = 0.03; see Table 2). Furthermore, compared to the 2-factor model, the 3-factor model showed significantly better model fit (χ^2^ [*df* = 9] = 181.328, *p* < 0.001; Table 2), and the 3-factor solution fit the hypothesized financial hardship domains. While the four-factor solution showed a significantly better fit than the 3-factor solution (χ^2^ [*df* = 8] = 77.24, *p* < 0.001), the additional factor was theoretically uninterpretable. Thus, the three-factor solution was more favorable due to its theoretical interpretability and parsimonious solution.

**Table 2:** Fit indices for the hypothesized models

SEM Model	χ^2^	*df*	RMSEA	CFI	TLI	SRMR
**Exploratory Factor Analysis (EFA; *N* = 2,243)**						
2-factor model	348.30 ^***^	34	.06	.97	.96	.06
3-factor model	126.36 ^***^	25	.04	.99	.98	.03
4-factor model	52.74 ^***^	17	.03	1.00	.99	.02
2-factor vs. 3-factor	181.51 ^***^	9	—	—	—	—
3-factor vs. 4-factor	78.42 ^***^	8	—	—	—	—
**Confirmatory Factor Analysis (CFA; *N* = 863)**						
1-factor measurement model	232.53 ^***^	43	.07	.96	.95	.07
3-factor measurement model	102.06 ^***^	40	.04	.99	.98	.04
Chi-Square test (1- vs. 3-factor measurement model)	79.94 ^***^	3	—	—	—	—
Second-order measurement model	Identical to the 3-factor measurement model					
**Domains of Financial Hardship and Inflammation (*N* = 863)**						
IL6 – MODEL 1	144.20 ^***^	64	.04	.99	.98	.04
IL6 – MODEL 2	193.72 ^***^	72	.04	.98	.98	.04
IL6 – MODEL 3	256.38 ^***^	88	.05	.98	.97	.04
CRP – MODEL 1	153.20 ^***^	64	.04	.98	.98	.04
CRP – MODEL 2	201.21 ^***^	72	.05	.98	.97	.04
CRP – MODEL 3	263.46 ^***^	88	.05	.98	.97	.04
Fibrinogen – MODEL 1	144.44 ^***^	64	.04	.99	.98	.04
Fibrinogen – MODEL 2	194.22 ^***^	72	.04	.98	.98	.04
Fibrinogen – MODEL 3	256.56 ^***^	88	.05	.98	.97	.04
**General Financial Hardship and Inflammation (*N* = 863)**						
IL6 – MODEL 1	179.43 ^***^	70	.04	.98	.98	.05
IL6 – MODEL 2	237.61 ^***^	80	.05	.98	.97	.05
IL6 – MODEL 3	417.30 ^***^	100	.06	.96	.95	.06
CRP – MODEL 1	184.02 ^***^	70	.04	.98	.97	.05
CRP – MODEL 2	240.10 ^***^	80	.05	.98	.97	.05
CRP – MODEL 3	421.51 ^***^	100	.06	.96	.95	.06
Fibrinogen – MODEL 1	174.45 ^***^	70	.04	.98	.98	.05
Fibrinogen – MODEL 2	233.56 ^***^	80	.05	.98	.97	.05
Fibrinogen – MODEL 3	414.79 ^***^	100	.06	.96	.95	.06

*χ*^*2*^ Chi-square test of model fit, *df* degree of freedom for the chi-square test of model fit, *RMSEA* Root mean square error of approximation, *CFI* Comparative fit index, *TLI* Tucker–Lewis index, *SRMR* Standardized root mean squared residual, *MODEL 1* Adjusted for age (years), sex (0 = female, 1 = male), *MODEL 2* Adjusted for age (years), sex (0 = female, 1 = male), and BMI (kg/m^2^), *MODEL 3* Adjusted for age (years), sex (0 = female, 1 = male), BMI (kg/m^2^), education (0 = no bachelor’s degree, 1 = bachelor’s degree or higher), and race (0 = racial/ethnic minorities, 1 = non-Hispanic white)

*** = *p* < .001

We examined additional factor retention criteria to see the robustness of the 3-factor solution. While the findings were mixed, the results from parallel analysis based on the 95th percentile of random eigenvalues and comparison data suggested the three-factor solution. Other criteria suggested one (Hull method) and two (Empirical Kaiser criterion) as the recommended number of factors. Given this additional finding, we have enough empirical support for the hypothesized three-factor model of financial hardship. Detailed information regarding other factor retention criteria is presented in Supplementary Material 2.

The rotated factor loadings for the 2-factor, 3-factor, and 4-factor models are presented in Supplementary Material 3. The rotated factor loadings for the 3-factor model supported the hypothesized material-psychological-behavioral framework of financial hardship. Income to poverty line ratio, health insurance coverage, and public/governmental financial assistance status showed higher factor loadings on the material domain. Perceived financial situation and control and perceived financial strains showed higher factor loadings in the psychological domain. Finally, all behavioral-related items showed higher factor loadings in the behavioral domain. The 3-factor solution showed no negative residual variance (Supplementary Material 3). The factor correlations for the 3-factor solution range from 0.33 to 0.47 (Supplementary Material 3). Finally, the alpha and omega scores indicate sufficient reliability (see Supplementary Material 4).

### Confirmatory Factor Analysis (CFA)

CFA was conducted to see how well the three-factor measurement model of financial hardship in a different sample was drawn from the MIDUS Refresher study. Model modification indices from the initial analysis suggested correlating missed credit card payments and other debt payments (e.g., car/student loan). The final three-factor measurement model fulfilled overall goodness-of-fit criteria (χ^2^ = 102.06, *df* = 40; RMSEA = 0.04; CFI = 0.99; TLI = 0.98; SRMR = 0.04; Table 2). We run a rival one-factor measurement model correlating missed credit card payments and other debt payments (see Table 2 for the model fit of the rival model). A significant decrement in fit was observed when comparing the hypothesized three-factor measurement model to the one-factor model (Δχ^2^ = 79.94, *df* = 3, *p* < 0.001). Thus, the three-factor model of financial hardship fit well with data from a different sample of adults, and it showed a significantly better fit than the one-factor measurement model. Standardized parameter estimates from the final three-factor measurement model ranged from 0.56 to 0.86 (Supplementary Material 5). We also tested the second-order measurement model of financial hardship with three latent first-order factors (i.e., material, psychological, and behavioral). As expected, the model fit of the second-order measurement model was identical to the three-factor measurement model (see Supplementary Material 5 for standardized parameter estimates).

### Financial hardship and inflammation

We tested the association between financial hardship and inflammation markers in two different ways. First, we tested the association between domains of financial hardship and inflammation markers by regressing each inflammation marker on the three-factor measurement model of financial hardship. Second, we examined the association between the second-order general latent factor of financial hardship and each inflammation marker.

### Domains of financial hardship and inflammation

We run structural equation models to examine the association between financial hardship domains and each inflammation marker. All the models fulfilled the overall goodness-of-fit criteria (see Table 2). Detailed results on the association between financial hardship and inflammation markers are presented in Table 3 (see Supplementary Material 6 for figure representations).

**Table 3:** Findings from structural equation models on the association between domains of financial hardship and inflammation (*N* = 863)

Predictor	MODEL 1		MODEL 2		MODEL 3	
	Estimate (*SE*)	95%*CI*	Estimate (*SE*)	95%*CI*	Estimate (*SE*)	95%*CI*
**Domain of Financial Hardship and IL6**						
Material domain	0.43 (0.11) ^***^	**[0.25, 0.62]**	0.38 (0.11) ^***^	**[0.20, 0.56]**	0.46 (0.21) ^*^	**[0.11, 0.81]**
Psychosocial domain	-0.22 (0.12) ^†^	[-0.41, -0.03]	-0.19 (0.04) ^†^	[-0.36, -0.01]	-0.21 (0.14)	[-0.43, -0.02]
Behavioral domain	0.19 (0.11) ^†^	[0.01, 0.36]	0.14 (0.11)	[0.04, 0.31]	-0.12 (0.11)	[0.06, 0.31]
Age	0.49 (0.04) ^***^	**[0.42, 0.56]**	0.47 (0.04) ^***^	**[0.41, 0.53]**	0.48 (0.04) ^***^	**[0.41, 0.55]**
Sex (0 = female, 1 = male)	0.04 (0.04)	[-0.02, 0.09]	0.04 (0.03)	[-0.01, 0.09]	0.04 (0.03)	[-0.01, 0.10]
Body mass index			0.33 (0.02) ^***^	**[0.30, 0.36]**	0.33 (0.02) ^***^	**[0.30, 0.37]**
Education (0 = lower, 1 = bachelor’s or higher)					0.08 (0.07)	[-0.04, 0.20]
Race (0 = minorities, 1 = NH white)					0.01 (0.07)	[-0.10, 0.12]
**Domain of Financial Hardship and CRP**						
Material domain	0.22 (0.11) ^*^	**[0.05, 0.40]**	0.16 (0.10)	[-0.00, 0.33]	0.03 (0.18)	[-0.26, 0.33]
Psychosocial domain	-0.16 (0.11)	[-0.34, 0.03]	-0.12 (0.10)	[-0.29, 0.05]	-0.09 (0.12)	[-0.28, 0.11]
Behavioral domain	0.22 (0.11) ^*^	**[0.05, 0.40]**	0.16 (0.10)	[-0.01, 0.32]	0.18 (0.10) ^†^	[0.01, 0.35]
Age	0.25 (0.04) ^***^	**[0.18, 0.32]**	0.23 (0.04) ^*^	**[0.16, 0.29]**	0.22 (0.04) ^***^	**[0.15, 0.28]**
Sex (0 = female, 1 = male)	-0.12 (0.04) ^**^	**[-0.18, -0.06]**	-0.12 (0.03) ^***^	**[-0.17, -0.06]**	-0.12 (0.03) ^***^	**[-0.17, -0.07]**
Body mass index			0.39 (0.02) ^***^	**[0.36, 0.41]**	0.38 (0.01) ^***^	**[0.36, 0.41]**
Education (0 = lower, 1 = bachelor’s or higher)					-0.11 (0.06) ^†^	[-0.22, -0.00]
Race (0 = minorities, 1 = NH white)					-0.04 (0.06)	[-0.13, 0.05]
**Domain of Financial Hardship and Fibrinogen**						
Material domain	0.30 (0.11) ^**^	**[0.13, 0.48]**	0.20 (0.10) ^*^	**[0.04, 0.37]**	0.05 (0.18)	[-0.24, 0.34]
Psychosocial domain	-0.17 (0.11)	[-0.36, 0.02]	-0.08 (0.10)	[-0.24, 0.09]	-0.03 (0.11)	[-0.21, 0.16]
Behavioral domain	0.17 (0.10)	[-0.00, 0.33]	-0.09 (0.10)	[-0.07, 0.24]	0.11 (0.10)	[-0.06, 0.27]
Age	0.33 (0.04) ^***^	**[0.26, 0.39]**	0.29 (0.04) ^***^	**[0.24, 0.35]**	0.29 (0.04) ^***^	**[0.23, 0.35]**
Sex (0 = female, 1 = male)	-0.14 (0.04) ^***^	**[-0.19, -0.08]**	-0.13 (0.03) ^***^	**[-0.19, -0.08]**	-0.13 (0.03) ^***^	**[-0.18, -0.08]**
Body mass index			0.31 (0.02) ^***^	**[0.29, 0.34]**	0.31 (0.02) ^***^	**[0.28, 0.33]**
Education (0 = lower, 1 = bachelor’s or higher)					-0.08 (0.07)	[-0.19, 0.03]
Race (0 = minorities, 1 = NH white)					-0.10 (0.05) ^†^	[-0.19, -0.01]

MODEL 1 = adjusted for age, sex; MODEL 2 = adjusted for age, sex, and body mass index, MODEL 3 *=* adjusted for age, sex, body mass index, education, and race

95%*CI* = 95% confidence interval

† = *p* < .10

* = *p* < .05

** = *p* < .01

*** = *p* < .001

#### Domains of financial hardship and IL6

In the model adjusted for age and sex, IL6 was significantly associated with the material domain (Est = 0.43, *p* < 0.001, 95%CI = [0.25, 0.62]), but not psychological (Est = -0.22, *p* = 0.06, 95%CI = [-0.41, -0.03]) and behavioral (Est = 0.19, *p* = 0.09, 95%CI = [0.01, 0.19]) domains. Higher material hardship was associated with elevated IL6. The association between the material domain and IL6 remained significant after adding BMI, education, and race as additional covariates (Est = 0.46, *p* = 0.03, 95%CI = [0.11, 0.81]).

##### Domains of financial hardship and CRP

Adjusted for age and sex, CRP was significantly associated with both material (*Est* = 0.22, *p* = 0.036, 95%*CI* = [0.05, 0.40]) and behavioral (*Est* = 0.22, *p* = 0.037, 95%*CI* = [0.05, 0.40]), but not psychological domain (*Est* = -0.16, *p* = 0.16, 95%*CI* = [-0.34, 0.03]). Higher material and behavioral financial hardship were associated with more elevated CRP. However, the associations became non-significant for both material (*Est* = 0.03, *p* = 0.85, 95%*CI* = [-0.26, 0.33]) and behavioral (*Est* = 0.18, *p* = 0.08, 95%*CI* = [0.01, 0.35]) domains after BMI, education, and race added as additional covariates.

##### Domains of Financial Hardship and Fibrinogen

The material domain (*Est* = 0.25, *p* = 0.014, 95%*CI* = [0.08, 0.42]), but not psychological (*Est* = -0.11, *p* = 0.31, 95%*CI* = [-0.28, 0.07]) and behavior domains (*Est* = 0.14, *p* = 0.17, 95%*CI* = [-0.03, 0.30]) of financial hardship, was significantly associated with fibrinogen in the model adjusted for age and sex. Higher material financial hardship was associated with more elevated fibrinogen. The material domain remained significantly associated with fibrinogen after adding BMI as an additional covariate (*Est* = 0.20, *p* = 0.04, 95%*CI* = [0.04, 0.37]). However, the material domain was not significantly associated with fibrinogen in the fully adjusted model (*Est* = 0.05, *p* = 0.77, 95%*CI* = [-0.24, 0.34]).

### General latent factor of financial hardship and inflammation

**Fig. 1:**
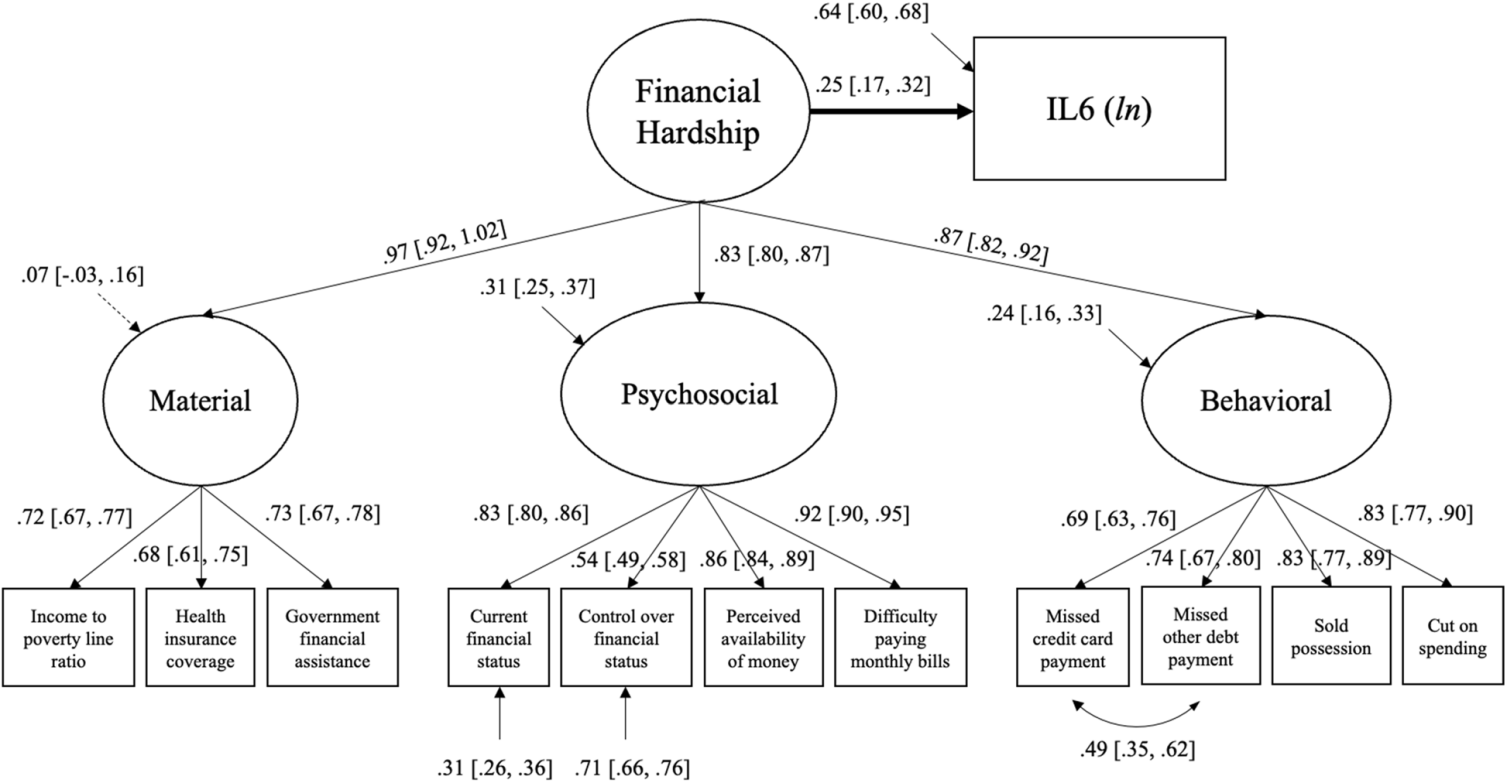
The association between the general latent factor of financial hardship and IL6, adjusted for age, sex, BMI, education, and race (*N* = 863). Straight lines represent significant estimates. Estimates indicate standardized estimates with a 95% confidence interval. Circles represent latent variables, and squares represent observed variables

**Fig. 2:**
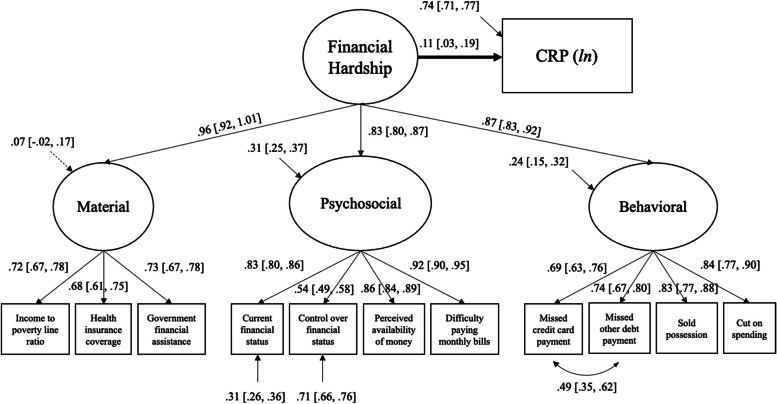
The association between the general latent factor of financial hardship and CRP, adjusted for age, sex, BMI, education, and race (*N* = 863). Straight lines represent significant estimates. Estimates indicate standardized estimates with 95% confidence intervals. Circles represent latent variables, and squares represent observed variables

**Fig. 3:**
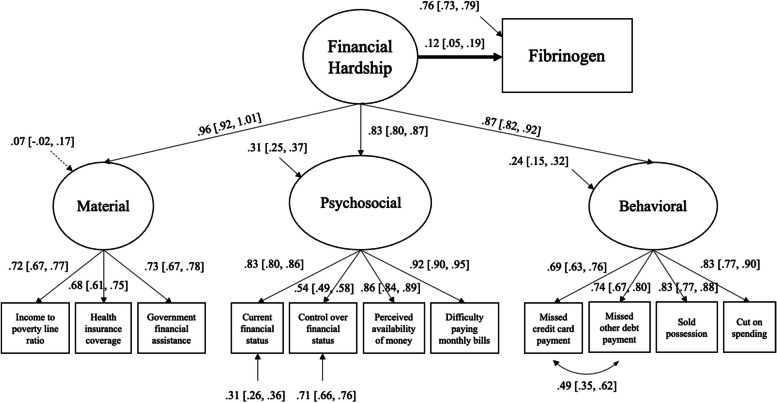
The association between the general latent factor of financial hardship and CRP, adjusted for age, sex, BMI, education, and race (*N* = 863). Straight lines represent significant estimates. Estimates indicate standardized estimates with 95% confidence intervals. Circles represent latent variables, and squares represent observed variables

All the structural equation models testing the association between the general latent factor of financial hardship and inflammation markers fulfilled the overall goodness-of-fit criteria (see Table 2). Detailed results on the association between financial hardship and inflammation markers are presented in Table 4 and Figs. 1, 2 and 3.

**Table 4:** Findings from structural equation models on the association between the general latent factor of financial hardship and inflammation (*N* = 863)

Predictor	MODEL 1		MODEL 2		MODEL 3	
	Estimate (*SE*)	95%*CI*	Estimate (*SE*)	95%*CI*	Estimate (*SE*)	95%*CI*
**General Financial Hardship and IL6**						
Financial hardship	0.34 (0.04) ^***^	**[0.28, 0.40]**	0.28 (0.04) ^***^	**[0.22, -0.34]**	0.25 (0.05) ^***^	**[0.17, 0.32]**
Age	0.44 (0.03) ^***^	**[0.39, 0.50]**	0.44 (0.03) ^***^	**[0.39, 0.48]**	0.44 (0.03) ^***^	**[0.40, 0.49]**
Sex (0 = female, 1 = male)	0.01 (0.03)	[-0.04, 0.07]	0.02 (0.03)	[-0.02, 0.07]	0.03 (0.03)	[-0.02, 0.08]
Body mass index			0.35 (0.02) ^***^	**[0.32, 0.38]**	0.34 (0.02) ^***^	**[0.31, 0.37]**
Education (0 = lower, 1 = bachelor’s or higher)					-0.01 (0.04)	[-0.07, 0.05]
Race (0 = minorities, 1 = NH white)					-0.09 (0.03) ^**^	**[-0.14, -0.04]**
**General Financial Hardship and CRP**						
Financial hardship	0.24 (0.04) ^***^	**[0.17, 0.31]**	0.17 (0.04) ^***^	**[0.10, 0.23]**	0.11 (0.05) ^*^	**[0.03, 0.19]**
Age	0.21 (0.04) ^***^	**[0.15, 0.27]**	0.20 (0.03) ^***^	**[0.15, 0.25]**	0.19 (0.03) ^***^	**[0.14, 0.25]**
Sex (0 = female, 1 = male)	-0.14 (0.03) ^***^	**[-0.19, -0.08]**	-0.13 (0.03) ^***^	**[-0.18, -0.08]**	-0.13 (0.03) ^***^	**[-0.18, -0.08]**
Body mass index			0.40 (0.01) ^***^	**[0.38, 0.42]**	0.39 (0.01) ^***^	**[0.27, 0.41]**
Education (0 = lower, 1 = bachelor’s or higher)					-0.10 (0.04) ^**^	**[-0.15, -0.04]**
Race (0 = minorities, 1 = NH white)					-0.04 (0.03)	[-0.09, 0.01]
**General Financial Hardship and Fibrinogen**						
Financial hardship	0.24 (0.04) ^***^	**[0.18, 0.30]**	0.18 (0.04) ^***^	**[0.12, 0.24]**	0.12 (0.05) ^**^	**[0.05, 0.19]**
Age	0.29 (0.03) ^***^	**[0.23, 0.34]**	0.28 (0.03) ^***^	**[0.23, 0.33]**	0.28 (0.03) ^***^	**[0.23, 0.33]**
Sex (0 = female, 1 = male)	-0.15 (0.03) ^***^	**[-0.20, -0.10]**	-0.14 (0.03) ^***^	**[-0.19, -0.09]**	-0.14 (0.03) ^***^	**[-0.19, -0.09]**
Body mass index			0.32 (0.01) ^***^	**[0.30, 0.34]**	0.31 (0.01) ^***^	**[0.29, 0.34]**
Education (0 = lower, 1 = bachelor’s or higher)					-0.07 (0.04) ^*^	**[-0.14, -0.01]**
Race (0 = minorities, 1 = NH white)					-0.10 (0.03) ^**^	**[-0.15, -0.05]**

MODEL 1 = adjusted for age, sex; MODEL 2 = adjusted for age, sex, and body mass index, MODEL 3 *=* adjusted for age, sex, body mass index, education, and race

95%*CI* = 95% confidence interval

† = *p* < .10

* = *p* < .05

** = *p* < .01

*** = *p* < .001

### General financial hardship and IL6

Higher financial hardship was significantly associated with more elevated IL6 in the model adjusted for age and sex (*Est* = 0.34, *p* < 0.001, 95%*CI* = [0.28, 0.40]). The association between financial hardship and IL6 was attenuated after adding BMI but remained statistically significant (*Est* = 0.28, *p* < 0.001, 95%*CI* = [0.22, 0.34]). This association was further attenuated in the fully adjusted model (*Est* = 0.25, *p* < 0.001, 95%*CI* = [0.17, 0.32]; Fig. 1).

### General financial hardship and CRP

In the model adjusted for age and sex, higher financial hardship was associated with higher CRP (*Est* = 0.24, *p* < 0.001, 95%*CI* = [0.17, 0.31]). This association weakened but remained significant after adding BMI as an additional covariate (*Est* = 0.17, *p* < 0.001, 95%*CI* = [0.10, 0.23]). Finally, adding education and race into the model further attenuated the significant association between financial hardship and CRP (*Est* = 0.11, *p* = 0.017, 95%*CI* = [0.03, 0.19]; Fig. 2).

### General financial hardship and fibrinogen

Adjusted for age and sex, more financial hardship was associated with more elevated fibrinogen (*Est* = 0.24, *p* < 0.001, 95%*CI* = [0.18, 0.30]). Adding BMI as an additional covariate into the model attenuated the association between financial hardship and fibrinogen (*Est* = 0.18, *p* < 0.001, 95%*CI* = [0.12, 0.24]). Financial hardship remained significantly associated with fibrinogen in the fully adjusted model (*Est* = 0.12, *p* < 0.01, 95%*CI* = [0.05, 0.19]; Fig. 3).

## Discussion

Systemic inflammation has been shown to indicate future disease risk for several chronic conditions, including cardiovascular disease [14], cancer [15], diabetes [16, 17], and Alzheimer’s disease [18]. In addition, increasing evidence suggests an inverse association between socioeconomic circumstances and inflammatory processes. To further explicate socioeconomic correlates of systemic inflammation, this study investigated the association between material, psychological, and behavioral financial hardship indicators and inflammation markers among participants who completed the biomarker assessment in the MIDUS Refresher Study (2013–2016). We conceptualized financial hardship as a three-domain model including material, psychological, and behavioral domains [24]. We tested the construct validity of this conceptualization by investigating whether the items measuring financial hardship in the MIDUS Refresher Study captured these three domains. Our findings from our measurement model revealed that the measures of financial hardship for the hypothesized three-domain model used in our study captured an overarching construct of financial hardship. This indicates the general tendency for material, psychological, and behavioral dimensions of financial hardship to correlate. In addition, our results indicated that this multidimensional financial hardship was significantly associated with IL6, CRP, and fibrinogen.

### Measurement of financial hardship

Many terms are used in the research literature to describe the financial hardship experience, and no agreed-upon definitions or measures exist. An organizing framework from cancer prevention and survivorship [24, 26] suggests that the many terms used to measure financial hardship can be sorted into the material, psychological, and behavioral domains [49, 50]. We used that organizing framework in the current study, and our results showed that the items used to measure financial hardship in the MIDUS study could be sorted into those three domains to capture an overarching financial hardship construct. This is the first study, to our knowledge, that tests whether the three-domain model of financial hardship from cancer prevention and survivorship research is a valid operationalization of the financial hardship experience. Our results support this operationalization of financial hardship as a three-domain (material, psychological, and behavioral) model. Considering the material-psychological-behavioral framework of financial hardship is especially useful in health inequities research for multiple reasons. First, pathways regarding barriers to preventive behaviors caused by larger structural factors can be identified. For example, how residential segregation leads to resistance in health screening behavior can be further clarified by examining specific domains of financial barriers (e.g., material vs. psychological). Second, including concepts from each domain when studying financial hardship ensures that the particular socioeconomic environment can be targeted in intervention efforts to reduce health inequities. Tucker-Seeley and Thorpe Jr. [24] provide an example of how this framework can be applied in works related to intervention to address cancer health inequities.

### Socioeconomic circumstances and inflammation

Several studies have investigated the association between socioeconomic status and markers of inflammation [3, 9]. These studies have usually utilized traditional measures of socioeconomic status, such as educational attainment and household income, to operationalize socioeconomic circumstances. While most of these studies generally showed a statistically significant inverse association between socioeconomic status and levels of inflammation, the results have been equivocal depending on the indicator of SES, the marker of inflammation used, and the population studied. For example, in a study using data from the Multi-Ethnic Study of Atherosclerosis (MESA), a diverse cohort of adults 45–84 years of age in the US, Ranjit et al. (2007) showed that the association between CRP and education was only statistically significant among non-Hispanic white (NHW) and non-Hispanic black (NHB) respondents. Still, the association between income and CRP was statistically significant across all racial/ethnic groups (NWH, NHB, Hispanics, and Chinese) [5]. Yet data from the Atherosclerosis Risk in Communities (ARIC) study, a prospective study of men and women aged 45–64 at baseline from four US communities, found low educational attainment was statistically significantly associated with fibrinogen for white participants [9]. Results using data from the CARDIA study, a prospective study of white and black women and men aged 18–30 years recruited in the mid-1980s from four communities in the US, found that CRP and IL6 levels were statistically significantly inversely associated with educational attainment in each race/gender group except black men [51]. Future studies should test the intersectional impact of financial hardship and minoritized identity, especially race/ethnicity, on inflammation. This approach will help to elucidate the complexity of how social and structural factors lead to socioeconomic and racial health disparities.

### Financial hardship and inflammation

Few studies have investigated the association between financial hardship and markers of inflammation. Consistent with the research using traditional measures of socioeconomic status, the results from these studies also differ depending on the marker of inflammation used and the population studied. For example, Sturgeon and colleagues found that financial stress (operationalized by experiencing negative financial events) was statistically significantly associated with IL6, mediated by psychological well-being in a study of middle-aged adults between the ages of 40 and 65 years old in the Phoenix, AZ metro area [35]. However, Matthews et al. found that financial hardship was not statistically significantly associated with CRP or fibrinogen in a multi-site, community-based, prospective study of the menopause transition and aging [52].

Our results extend the research literature on the association between socioeconomic circumstances (including financial hardship) and inflammation in multiple ways. We showed that when examined separately, the material domain of financial hardship was consistently associated with IL6, CRP, and fibrinogen. We showed that BMI, education, or race did not fully explain the link between the material domain and IL6. On the other hand, the association between the material domain and CRP was fully explained by BMI. These results corroborate previous findings from Friedman and colleagues [53] that showed the consistent association between material deprivation (in their case, lowest income quartile) and IL6. Furthermore, they showed that the association between material deprivation and CRP was fully mediated by IL6, supporting the idea that IL6 stimulates the production of CRP [53]. Given that adipocytes are one of the main sources of IL6 [53], it made sense that BMI fully explained the link between the material domain and CRP. While the material domain showed relative importance in discriminating inflammation markers, future studies should prioritize testing if these findings replicated among racially and socioeconomically diverse samples and across different health outcomes (e.g., psychological well-being, major psychiatric disorders, and major chronic health outcomes). We also demonstrated that when utilized as an overarching latent construct based on the material-psychological-behavioral framework (i.e., the second-order measurement model), financial hardship showed a better prediction of IL6 and CRP. Our results investigating the association between a general latent financial hardship factor and multiple inflammation markers were robust as we included key sociodemographic, SES, and health-related covariates. Given the overwhelming representation of white participants from middle to higher levels of SES in the MIDUS study, replication among racially and socioeconomically diverse samples should be a priority for future research.

### Limitations

There are no agreed-upon definitions or measures of financial hardship across research fields. The selection of measures of financial hardship in this study was based on the availability of pre-collected MIDUS data. While we could show the validity of these measures in this study, there are limitations to some of the items we used. For example, we included the household income to poverty line ratio (adjusted for household size) as one of the measures of the material domain of financial hardship. Some might argue that this is similar to the commonly used SES measure. Adjusting for the poverty line based on household size may be a better way to capture material deprivation than just using raw measures of household income. However, future studies should focus on selecting a better measure of material deprivation, such as debt or net worth.

Similarly, we also included health insurance coverage as a proxy for medical care-related financial pressure. Better measures for this would be out-of-pocket expenses, debt, and decreased income due to medical care or treatment. Finally, items for the behavioral domain need to include a clear timeframe. Given that behavior may change across time, including time components (e.g., in the past month/past year) to the items may increase the ability to gauge the behavioral aspect of experiencing financial hardship. We also emphasize that our results may not generalize to the US population. While the MIDUS biomarker assessment sample is socio-demographically diverse, it is not nationally representative.

## Conclusion

The current study illustrates that multiple financial hardship aspects correlate with inflammatory processes. Further explicating factors in the socioeconomic environment to include indicators of financial hardship can help researchers better understand the pathway between socioeconomic status and the inflammation process. This analysis showed financial hardship measures' factor structure and validity that fit the material-psychological-material framework. Using the material-psychological-material framework, we also showed that financial hardship was associated with inflammation markers. Future work should explore race, gender, and age-stratified models to determine if the associations hold across socio-demographic groups. More specifically, research is needed on measuring financial hardship across the life course to better understand the association between financial hardship and age as a biological and social construct. In addition, further exploration of the implications of the accumulation of financial hardship over time on biological mechanisms, such as inflammatory processes on health across the life course, is needed. Such research could highlight the sensitive periods where financial hardship exerts its strongest influence on health across the life course.

### Supplementary Information

**Additional file 1.**

## Data Availability

Availability of data and materials The MIDUS datasets used in this analysis are available on the Inter-university Consortium for Political and Social Research (ICPSR) website: https://www.icpsr.umich.edu/web/ICPSR/series/203.
