# Supplementary material for: The association between material-psychological-behavioral framework of financial hardship and markers of inflammation: a cross-sectional study of the Midlife in the United States (MIDUS) Refresher cohort
Source: BMC Public Health. 2023 Sep 21;23:1845. doi: 10.1186/s12889-023-16745-x (PMC10514981; doi:10.1186/s12889-023-16745-x)
Supplement: Supplementary file 1 — Additional file 1. [file 12889_2023_16745_MOESM1_ESM.docx]

Supplementary Materials: The Association Between Material-Psychological-Behavioral Framework of Financial Hardship and Markers of Inflammation: A Cross-Sectional Study of the Midlife in the United States (MIDUS) Refresher Cohort

Agus Surachman, PhD^1,2^, Reginald Tucker-Seeley, ScD^3^, and David M. Almeida, PhD^4,5^

^1^Department of Epidemiology and Biostatistics, Dornsife School of Public Health, Drexel University, Philadelphia, USA

^2^College of Nursing and Health Professions, Drexel University, Philadelphia, USA

^3^ZERO – The End of Prostate Cancer, Alexandria, Virginia, USA

^4^Department of Human Development and Family Studies, The Pennsylvania State University, University Park, PA, USA

^5^Center for Healthy Aging, The Pennsylvania State University, University Park, PA, USA

**Corresponding Author:**

Agus Surachman, PhD

Dornsife School of Public Health, Drexel University

Philadelphia, PA, 19104

Email: [agus.surachman@drexel.edu](mailto:agus.surachman@drexel.edu)

**Table of Content**

[**Supplementary Material 1: Bivariate Polychoric Correlations**](#_Supplementary_Material_1:)

- *Supplementary Figure 1A*. Bivariate correlations among financial hardship items from the EFA sample (*N* = 2,243)
- *Supplementary Figure 1B*. Bivariate correlations among financial hardship items, inflammation markers, and covariates from the CFA sample (*N* = 863)

[**Supplementary Material 2: Additional Factor Retention Criteria in Exploratory Factor Analysis**](#_Supplementary_Material_2:)

- *Supplementary Figure 2A*. Scree plot from the parallel analysis. The analysis indicated that the suggested number of factors was three.
- *Supplementary Figure 2B*. Plot from the comparison data method. The analysis indicated that the suggested number of factors was three.
- *Supplementary Figure 2C*. Scree plot from the Empirical Kaiser criterion method. The analysis indicated that the suggested number of factors was two.

[**Supplementary Material 3: Results from Exploratory Factor Analysis**](#_Supplementary_Material_3:)

- *Supplementary Table 1A*. CF-Facparsim (oblique) rotated loadings from exploratory factor analysis for the 2-factor solution of financial hardship measures (*N* = 2,243)
- *Supplementary Table 1B*. CF-Facparsim (oblique) rotated loadings from exploratory factor analysis for the 3-factor solution of financial hardship measures (*N* = 2,243)
- Supplementary Table *1C*. CF-Facparsim (oblique) rotated loadings from exploratory factor analysis for the 2-factor solution of financial hardship measures (*N* = 2,243)

[**Supplementary Material 4: Reliability Information**](#_Supplementary_Material_4:)

- *Supplementary Table 2A*. Overall reliability indicators
- *Supplementary Table 2B*. Reliability indicators for subscales

[**Supplementary Material 5: Results from Confirmatory Factor Analyses**](#_Supplementary_Material_5:)

- *Supplementary Table* 3. Detailed findings from confirmatory factor analyses for the three-factor measurement and second-order measurement models of financial hardship (*N* = 863)

[**Supplementary Material 6: Figure Representations of the Association Between Financial Hardship and Inflammation**](#_Supplementary_Material_6:)

- *Supplementary Figure 3A*. The figure represents the association between domains of financial hardship and IL6, adjusted for age, sex, BMI, education, and race.
- *Supplementary Figure 3B*. The figure represents the association between domains of financial hardship and IL6, adjusted for age, sex, BMI, education, and race.
- *Supplementary Figure 3C*. The figure represents the association between the general latent factor of financial hardship and IL6, adjusted for age, sex, BMI, education, and race.
- *Supplementary Figure 3D*. Figure representation of the association between the general latent factor of financial hardship and CRP, adjusted for age, sex, BMI, education, and race.

# **Supplementary Material 1: Bivariate Polychoric Correlations**

We used the mixedCor function from the psych package in R to draw bivariate correlations among financial hardship measures and inflammation using the EFA (Supplementary Figure 1A) and CFA sample (Supplementary Figure 1B). The description of the labels in the figures is presented in Supplementary Table 1.


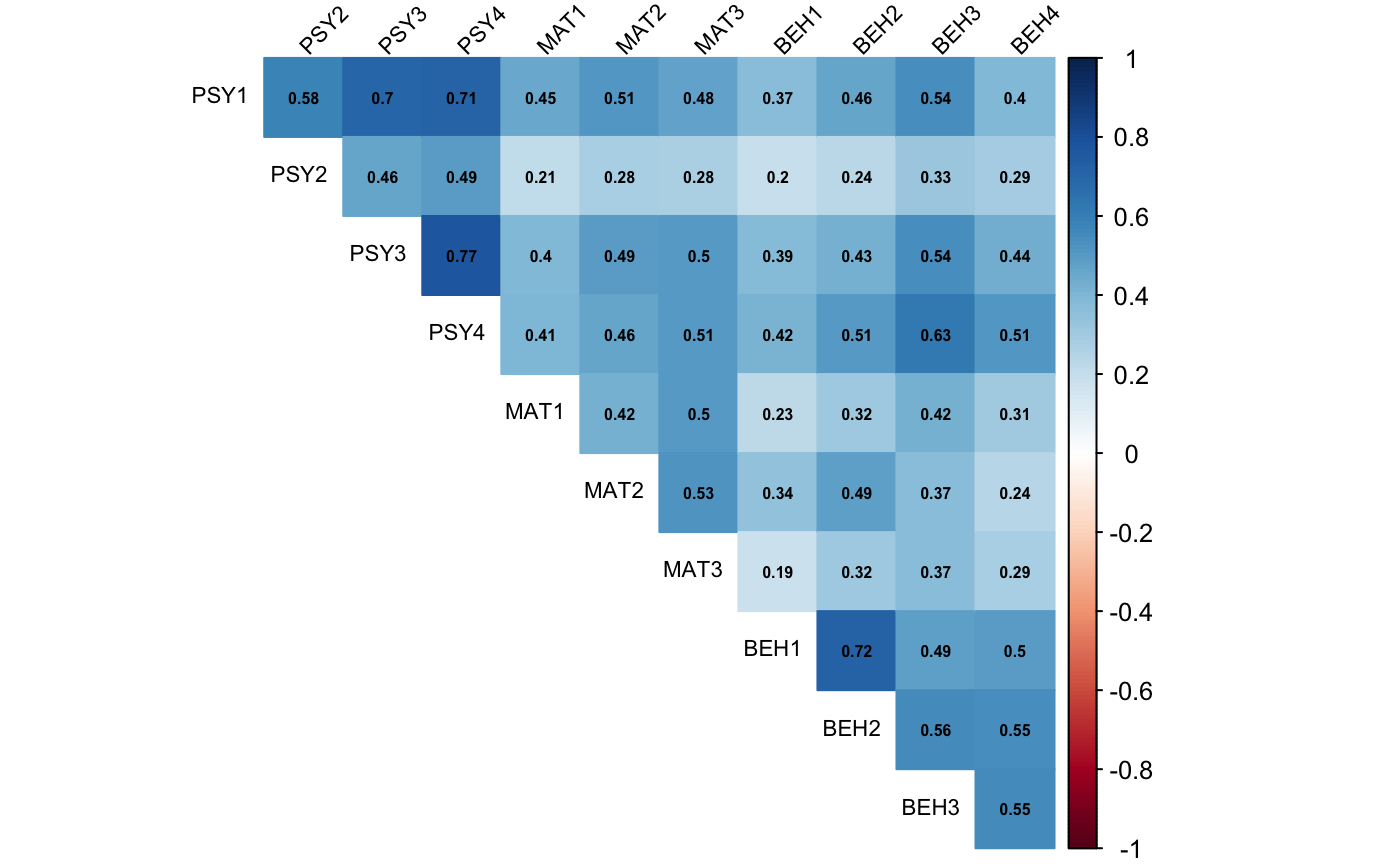


*Supplementary Figure 1A*. Bivariate correlations among financial hardship items from the EFA sample (*N* = 2,243)


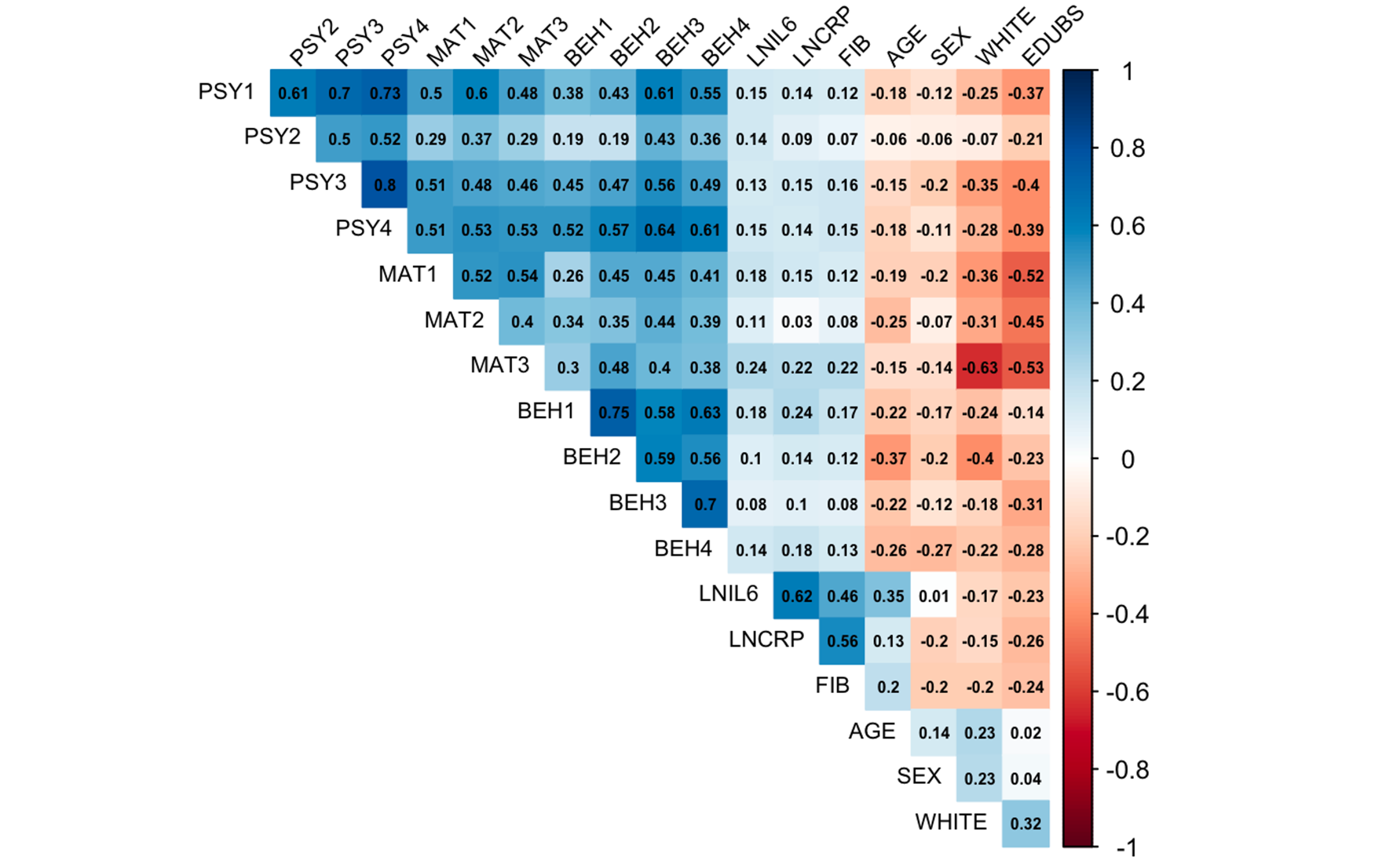


*Supplementary Figure 1B*. Bivariate correlations among financial hardship items, inflammation markers, and covariates from the CFA sample (*N* = 863)

Supplementary Table 1

Description of the label on the bivariate correlation figures

| Label | Description |
| --- | --- |
| PSY1 | Perceived current financial situation |
| PSY2 | Perceived financial control |
| PSY3 | Perceived Availability of money to meet needs |
| PSY4 | Perceived difficulty paying monthly bills |
| MAT1 | Income to poverty line ratio |
| MAT2 | Health insurance coverage |
| MAT3 | Public/government financial assistance |
| BEH1 | Missed a credit card payment |
| BEH2 | Missed other debt payment |
| BEH3 | Sold possessions to make ends meet |
| BEH4 | Cut back on spending |
| LNIL6 | Interleukin 6 (*ln*) |
| LNCRP | C-reactive protein (*ln*) |
| FIB | Fibrinogen |
| AGE | Age (years) |
| SEX | Sex (0 = female, 1 = male) |
| WHITE | Race (0 = racial/ethnic minorities, 1 = non-Hispanic White) |
| EDUBS | Education (0 = lower, 1 = bachelor’s degree or higher) |

# **Supplementary Material 2: Additional Factor Retention Criteria in Exploratory Factor Analysis**

We examined additional factor retention criteria to see the robustness of the 3-factor solution, including parallel analysis based on the 95th percentile of random eigenvalues, comparison data, Kaiser-Guttman criterion, Lower bound of RMSEA 90% confidence interval, Hull method, Empirical Kaiser criterion, and sequential chi-squared model test and Akaike information criterion. We examined these additional factor retention criteria in R.

1. **Parallel Analysis Based on the 95th Percentile of Random Eigenvalues**

Since parallel analysis in Mplus is only available for continuous variables, we conducted this analysis in R using the *fa.parallel* function from the *psych* package (Revelle, 2017). We utilized the polychoric correlation matrix in the analysis. We used the weighted least squares as the factor method: eigenvalues were based on factor analysis and included 1,000 simulated analyses. The parallel analysis showed that the suggested number of factors was three.


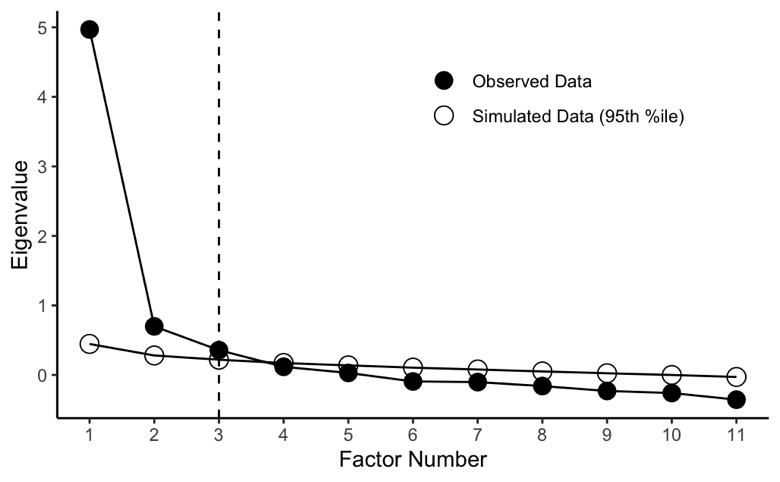


*Supplementary Figure 2A*. Scree plot from the parallel analysis. The analysis indicated that the suggested number of factors was three.

1. **Comparison Data**

We used the *CD* function from the *EFAtools* package (Steiner & Grieder, 2020) to examine the comparison data method. The *CD* function required raw data in the model. We used the *lavCor* function to specify that variables with four categories or less as ordinal variables. We specified four as the maximum number of factors to extract to match the analysis in Mplus. Alpha was specified at .4. Comparison data showed that the suggested number of factors was three.


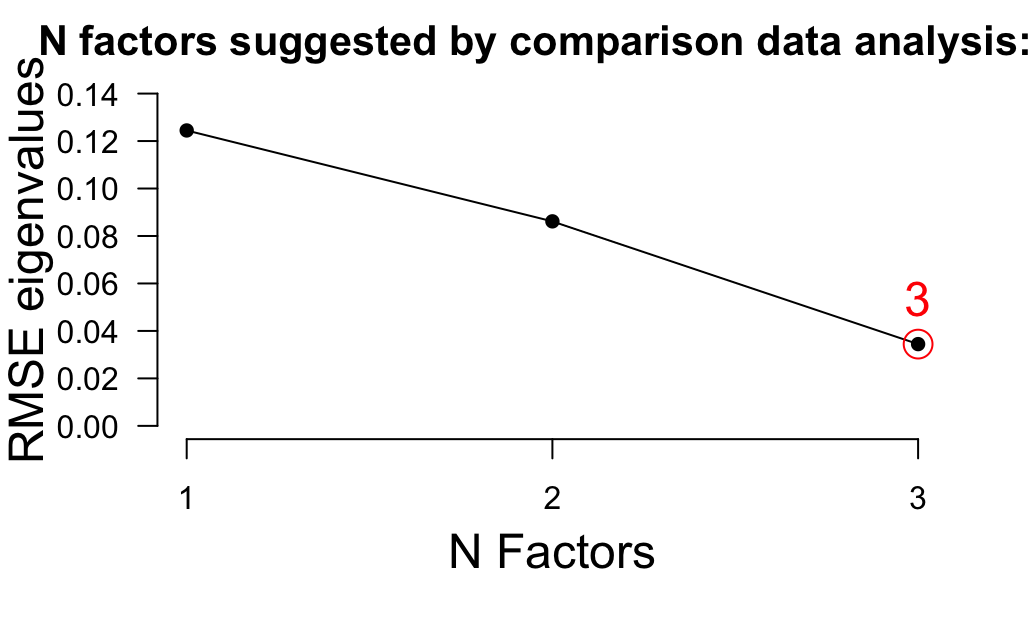


*Supplementary Figure 2B*. Plot from the comparison data method. The analysis indicated that the suggested number of factors was three.

1. **Hull Method**

We also used a function from the *EFAtools* package (Steiner & Grieder, 2020) to get the suggested factor number using the Hull method. Hull analysis performed testing 0 to 4 factors using unweighted least squares (ULS) as the estimation method and used the 90% eigenvalues as the rule to determine the number of factors. The hull method suggested a one-factor solution.

1. **Empirical Kaiser Criterion**

The empirical Kaiser criterion was examined using the *EKC* function from the *EFAtools* package (Steiner & Grieder, 2020). We used the polychoric correlation matrix in the analysis. The suggested number of factors was two.


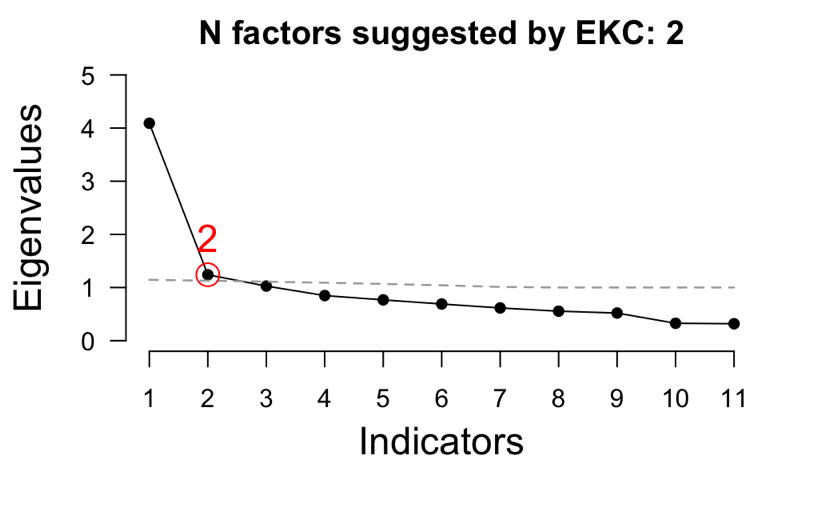


*Supplementary Figure 2C*. Scree plot from the Empirical Kaiser criterion method. The analysis indicated that the suggested number of factors was two.

1. **Summary**

Based on the four additional factor retention criteria, two criteria suggested a three-factor solution (Parallel Analysis and Comparison Data), while the other two criteria suggested one (Hull Method) and two (Empirical Kaiser criterion) as the suggested number of factors. Despite the mixed results, we firmly believe that we have sufficient support to conclude that the three were the recommended number of factors.

References

Revelle, W. R. (2017). psych: Procedures for psychological, psychometric, and personality research. Northwestern University, Evanston, Illinois. R package version 2.3.3, [https://CRAN.R-project.org/package=psych](https://cran.r-project.org/package=psych).

Steiner, M. D., & Grieder, S. (2020). EFAtools: An R package with fast and flexible implementations of exploratory factor analysis tools. *Journal of Open Source Software*, *5*(53), 2521.

Crawford, A. V., Green, S. B., Levy, R., Lo, W. J., Scott, L., Svetina, D., & Thompson, M. S. (2010). Evaluation of parallel analysis methods for determining the number of factors. *Educational and Psychological Measurement*, *70*(6), 885-901.

# **Supplementary Material 3: Results from Exploratory Factor Analysis**

*Supplementary Table 1A*. CF-Facparsim (oblique) rotated loadings from exploratory factor analysis for the 2-factor solution of financial hardship measures (*N* = 2,243)

| **CF-Facparsim (oblique) Rotated Factor Loadings for Two-Class Model (cut off = .4)** | | | | |
| --- | --- | --- | --- | --- |
| Items | Factor 1 | Factor 2 | Residual Variance | |
| Income to poverty line ratio | **0.52^*^** | 0.16^*^ | 0.63 | |
| Health insurance coverage | 0.38^*^ | 0.24^*^ | 0.72 | |
| Public/government financial assistance | **0.41^*^** | 0.30^*^ | 0.64 | |
| Perceived current financial situation | **0.73^*^** | 0.18^*^ | 0.32 | |
| Perceived control over financial situation | **0.53^*^** | 0.06^*^ | 0.69 | |
| Perceived availability of money | **0.75^*^** | 0.22^*^ | 0.25 | |
| Perceived difficulty paying bills | **0.69^*^** | 0.32^*^ | 0.22 | |
| Missed a credit card payment | -0.11^*^ | **0.84^*^** | 0.37 | |
| Missed other debt payment | -0.06 | **0.93^*^** | 0.19 | |
| Sold possessions to make ends meet | 0.31^*^ | **0.54^*^** | 0.47 | |
| Cut back on spending | 0.17^*^ | **0.57^*^** | 0.56 | |
| **CF-Facparsim (oblique) Factor Correlations for Two-Class Model** | | | | |
| Factor 1 | 1 |  |  |  |
| Factor 2 | .43^*^ | 1 |  |  |

*Note*: * = significant at *p* < .05

*Supplementary Table 1B*. CF-Facparsim (oblique) rotated loadings from exploratory factor analysis for the 3-factor solution of financial hardship measures (*N* = 2,243)

| **CF-Facparsim (oblique) Rotated Factor Loadings (cut off = .4)** | | | | |
| --- | --- | --- | --- | --- |
| Items | Factor 1: Material Domain | Factor 2: Psychosocial Domain | Factor 3: Behavioral Domain | Residual variance |
| Income to poverty line ratio | **.76^*^** | .06 | -.05 | .40 |
| Health insurance coverage | **.60^*^** | -.00 | .09 | .59 |
| Public/government financial assistance | **.62^*^** | .02 | .16**^*^** | .51 |
| Perceived current financial situation | .31**^*^** | **.62^*^** | .04 | .32 |
| Perceived control over financial situation | .08 | **.57^*^** | -.03 | .64 |
| Perceived availability of money | .31**^*^** | **.64^*^** | .08**^*^** | .26 |
| Perceived difficulty paying bills | .25**^*^** | **.66^*^** | .18**^*^** | .20 |
| Missed a credit card payment | .01 | .07 | **.78^*^** | .35 |
| Missed other debt payment | .21**^*^** | -.02 | **.82^*^** | .19 |
| Sold possessions to make ends meet | .20**^*^** | .32**^*^** | **.44^*^** | .47 |
| Cut back on spending | .03 | .30**^*^** | **.50^*^** | .54 |
| **CF-Facparsim (oblique) Factor Correlations** | | | | |
| Factor 1: Psychological domain | 1 |  |  |  |
| Factor 2: Material domain | .47^*^ | 1 |  |  |
| Factor 3: Behavioral domain | .33^*^ | .34^*^ | 1 |  |

*Note*: * = significant at *p* < .05

Supplementary Table *1C*. CF-Facparsim (oblique) rotated loadings from exploratory factor analysis for the 2-factor solution of financial hardship measures (*N* = 2,243)

| **CF-Facparsim (oblique) Rotated Factor Loadings for Four-Class Model (cut off = .4)** | | | | | |
| --- | --- | --- | --- | --- | --- |
| Items | Factor 1 | Factor 2 | Factor 3 | Factor 4 | Residual Variance |
| Income to poverty line ratio | **0.67^*^** | 0.10^*^ | -0.07 | 0.14^*^ | 0.41 |
| Health insurance coverage | **0.54^*^** | -0.00 | 0.04 | 0.17^*^ | 0.59 |
| Public/government financial assistance | **0.58^*^** | 0.20^*^ | 0.21^*^ | -0.07 | 0.46 |
| Perceived current financial situation | 0.15^*^ | **0.75^*^** | 0.06^*^ | 0.10^*^ | 0.20 |
| Perceived control over financial situation | -0.04 | **0.61^*^** | -0.02 | 0.10 | 0.57 |
| Perceived availability of money | 0.21^*^ | 0.38^*^ | -0.04 | **0.47^*^** | 0.28 |
| Perceived difficulty paying bills | 0.15^*^ | 0.30^*^ | -0.02 | **0.64^*^** | 0.17 |
| Missed a credit card payment | -0.02 | 0.08 | **0.70^*^** | 0.17^*^ | 0.36 |
| Missed other debt payment | 0.17^*^ | 0.07^*^ | **0.79^*^** | 0.10^*^ | 0.16 |
| Sold possessions to make ends meet | 0.13^*^ | 0.04 | 0.26^*^ | **0.53^*^** | 0.43 |
| Cut back on spending | -0.02 | 0.09^*^ | 0.35^*^ | **0.43^*^** | 0.53 |
| **CF-Facparsim (oblique) Factor Correlations for Two-Class Model** | | | |  |  |
| Factor 1 | 1 |  |  |  |  |
| Factor 2 | .40^*^ | 1 |  |  |  |
| Factor 3 | .23^*^ | .19^*^ | 1 |  |  |
| Factor 4 | .40^*^ | .54^*^ | .40^*^ | 1 |  |

*Note*: * = significant at *p* < .05

# **Supplementary Material 4: Reliability**

We used the omega function from the psych package to calculate reliability indicators from both the EFA and CFA samples. The information is summarized below.

*Supplementary Table 2A*. Overall reliability indicators

| Overall Reliability Indicator | Value |  |
| --- | --- | --- |
|  | EFA Sample (*N* = 2,243) | CFA Sample (*N* = 863) |
| Alpha | .90 | .91 |
| G.6 | .91 | .93 |
| Omega Hierarchical | .75 | .80 |
| Omega H asymptotic | .81 | .85 |
| Omega total | .93 | .94 |

*Supplementary Table 2B*. Reliability indicators for subscales

| Overall Reliability Indicator | EFA Sample | | | | CFA Sample | | | |
| --- | --- | --- | --- | --- | --- | --- | --- | --- |
|  | g | Material | Psychosocial | Behavioral | g | Material | Psychosocial | Behavioral |
| Omega total for total scores and subscales | .93 | .74 | .88 | .84 | .94 | .74 | .88 | .89 |
| Omega general for total scores and subscales | .75 | .45 | .71 | .44 | .80 | .55 | .65 | .59 |
| Omega group for total scores and subscales | .14 | .29 | .17 | .41 | .11 | .19 | .24 | .29 |

# **Supplementary Material 5: Results from Confirmatory Factor Analyses**

*Supplementary Table* 3. Detailed findings from confirmatory factor analyses for the three-factor measurement and second-order measurement models of financial hardship (*N* = 863)

|  | Estimate (*SE*) | 95%*CI* |
| --- | --- | --- |
| **Three-Factor Measurement Model** |  |  |
| **Material Domain** |  |  |
| Income to poverty line ratio | 0.73 (0.03) ^***^ | [0.68, 0.79] |
| Health insurance coverage | 0.71 (0.05) ^***^ | [0.63, 0.79] |
| Public/government financial assistance | 0.69 (0.04) ^***^ | [0.63, 0.76] |
| **Psychosocial Domain** |  |  |
| Perceived current financial situation | 0.83 (0.02) ^***^ | [0.80, 0.85] |
| Perceived financial control | 0.56 (0.03) ^***^ | [0.52, 0.60] |
| Perceived Availability of money to meet needs | 0.85 (0.02) ^***^ | [0.83, 0.88] |
| Perceived difficulty paying monthly bills | 0.93 (0.01) ^***^ | [0.91, 0.95] |
| **Behavioral Domain** |  |  |
| Missed a credit card payment | 0.65 (0.05) ^***^ | [0.58, 0.73] |
| Missed other debt payment | 0.71 (0.04) ^***^ | [0.65, 0.78] |
| Sold possessions to make ends meet | 0.86 (0.03) ^***^ | [0.81, 0.92] |
| Cut back on spending | 0.84 (0.04) ^***^ | [0.78, 0.90] |
| **Correlations Among Latent Domains** |  |  |
| Material – Psychosocial | 0.79 (0.03) ^***^ | [0.74, 0.84] |
| Material – Behavioral | 0.72 (0.04) ^***^ | [0.64, 0.79] |
| Psychosocial – Behavioral | 0.77 (0.03) ^***^ | [0.73, 0.82] |
| **Second Order Measurement Model** |  |  |
| **First Order: Material Domain** |  |  |
| Income to poverty line ratio | 0.73 (0.03) ^***^ | [0.68, 0.79] |
| Health insurance coverage | 0.71 (0.05) ^***^ | [0.63, 0.79] |
| Public/government financial assistance | 0.69 (0.04) ^***^ | [0.63, 0.76] |
| **First Order: Psychosocial Domain** |  |  |
| Perceived current financial situation | 0.83 (0.02) ^***^ | [0.80, 0.85] |
| Perceived financial control | 0.56 (0.03) ^***^ | [0.52, 0.60] |
| Perceived Availability of money to meet needs | 0.85 (0.02) ^***^ | [0.83, 0.88] |
| Perceived difficulty paying monthly bills | 0.93 (0.01) ^***^ | [0.91, 0.95] |
| **First Order: Behavioral Domain** |  |  |
| Missed a credit card payment | 0.65 (0.05) ^***^ | [0.58, 0.73] |
| Missed other debt payment | 0.71 (0.04) ^***^ | [0.65, 0.78] |
| Sold possessions to make ends meet | 0.86 (0.03) ^***^ | [0.81, 0.92] |
| Cut back on spending | 0.84 (0.04) ^***^ | [0.78, 0.90] |
| **Second Order: Financial Hardship** |  |  |
| Material domain | 0.85 (0.04) ^***^ | [0.80, 0.91] |
| Psychosocial domain | 0.93 (0.03) ^***^ | [0.88, 0.97] |
| Behavioral domain | 0.84 (0.03) ^***^ | [0.78, 0.89] |

*Note*: *** = significant at *p* < .001

# **Supplementary Material 6: Figure Representations of the Association Between Financial Hardship and Inflammation**

Below are figure representations of the association between financial hardship (domain and general latent factor) and inflammation markers.

*Supplementary Figure 3A*. The figure represents the association between domains of financial hardship and IL6, adjusted for age, sex, BMI, education, and race.

*Supplementary Figure 3B*. The figure represents the association between domains of financial hardship and CRP, adjusted for age, sex, BMI, education, and race.

*Supplementary Figure 3C*. The figure represents the association between domains of financial hardship and fibrinogen, adjusted for age, sex, BMI, education, and race.


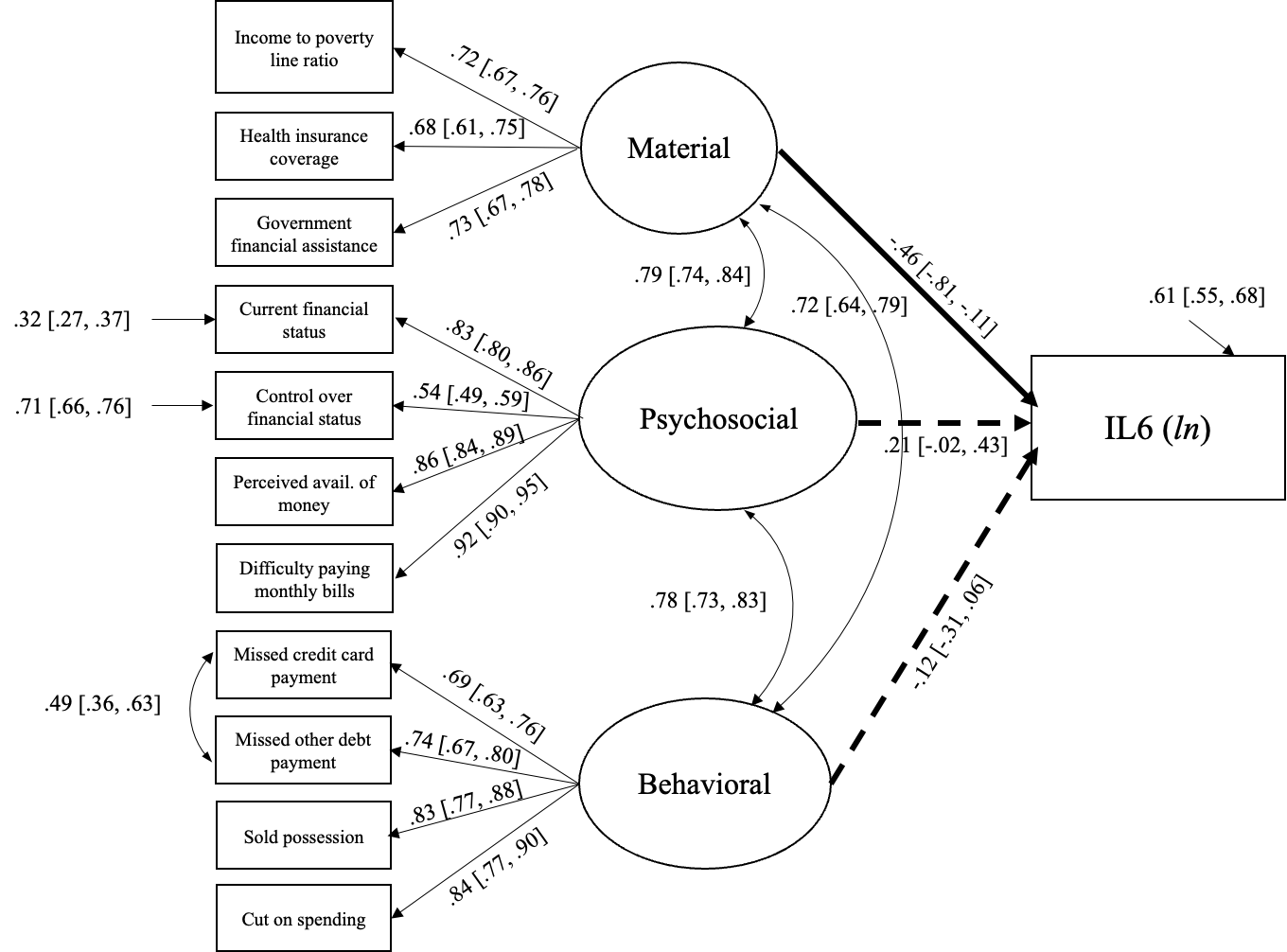


*Supplementary Figure 3A*. Figure representation of the association between domains of financial hardship and IL6, adjusted for age, sex, BMI, education, and race. Straight lines represent significant estimates, while dashed lines indicate non-significant estimates. Estimates indicate standardized estimates with 95% confidence intervals. Circles represent latent variables, and squares represent observed variables.


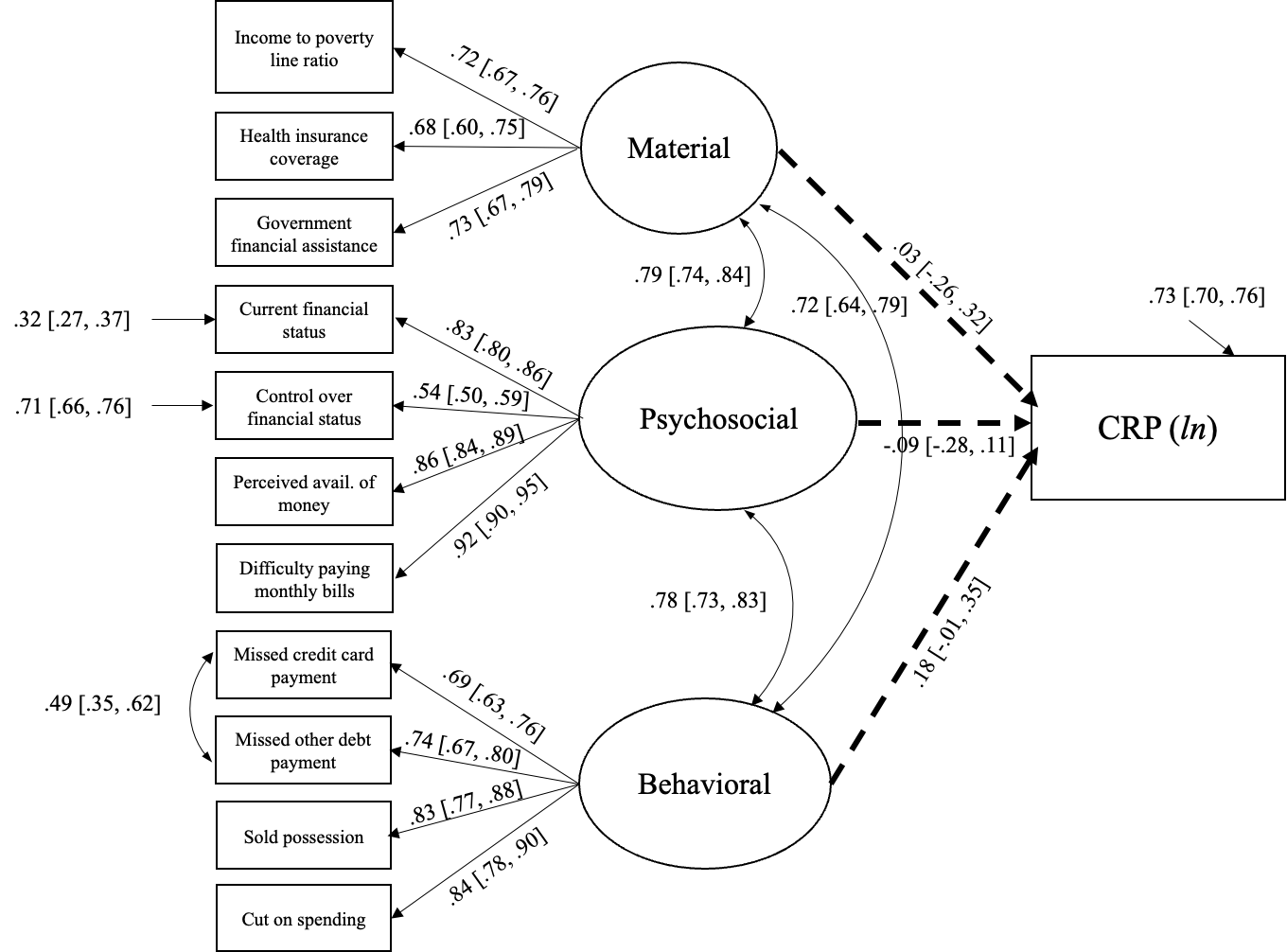


*Supplementary Figure 3B*. Figure representation of the association between domains of financial hardship and CRP, adjusted for age, sex, BMI, education, and race. Straight lines represent significant estimates, while dashed lines indicate non-significant estimates. Estimates indicate standardized estimates with 95% confidence intervals. Circles represent latent variables, and squares represent observed variables.


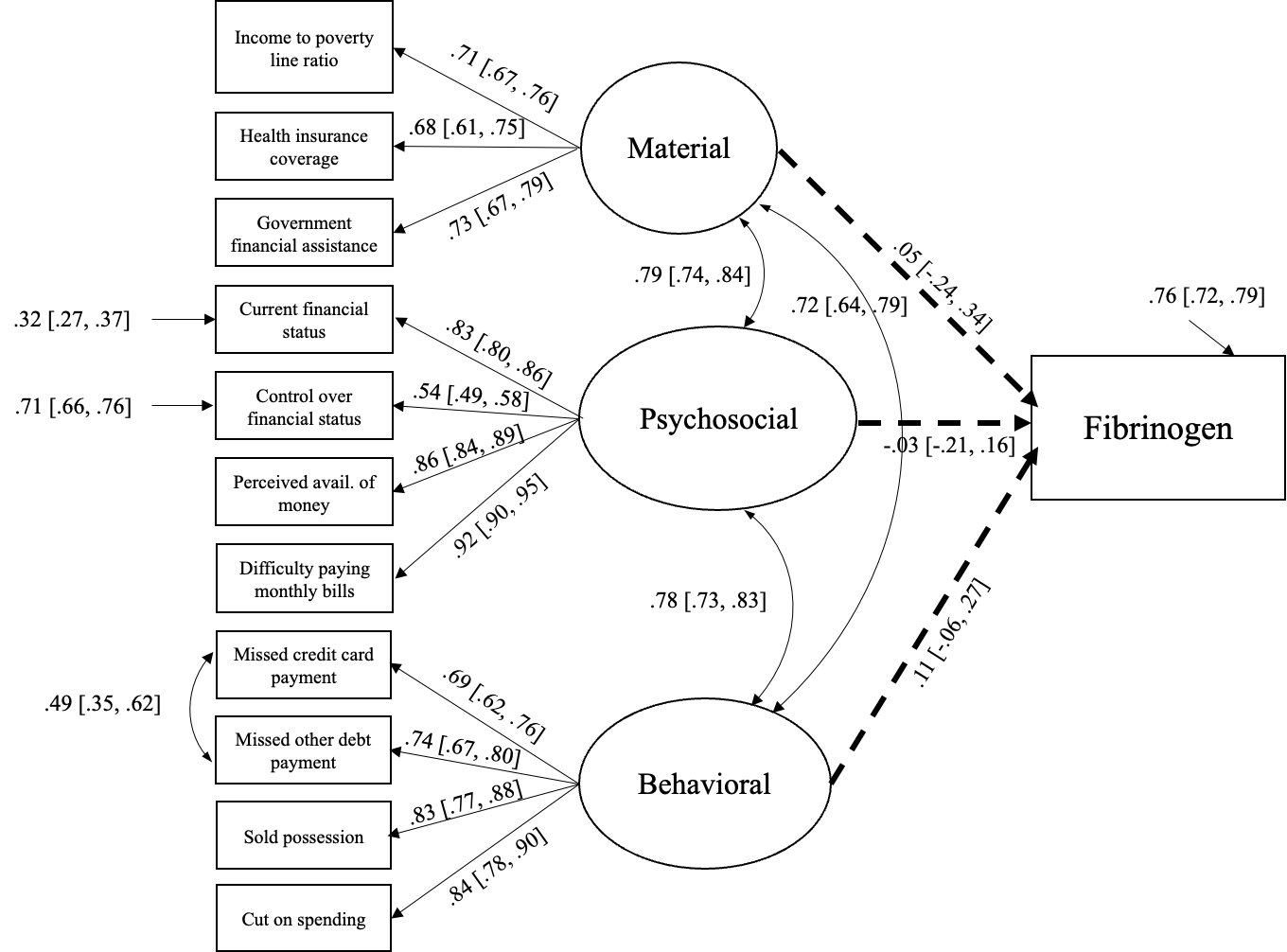


*Supplementary Figure 3C*. Figure representation of the association between domains of financial hardship and fibrinogen, adjusted for age, sex, BMI, education, and race. Straight lines represent significant estimates, while dashed lines indicate non-significant estimates. Estimates indicate standardized estimates with 95% confidence intervals. Circles represent latent variables, and squares represent observed variables.
